# PK11007 Covalently Inhibits Thioredoxin Reductase 1 to Induce Oxidative Stress and Autophagy Impairment in NSCLC Cells

**DOI:** 10.3390/antiox14101222

**Published:** 2025-10-11

**Authors:** Hanziyi Zhou, Shibo Sun, Haowen Liu, Tong Li, Yiran Xu, Rui Yang, Haiyan Liu, Leiyu He, Weiping Xu, Shui Guan, Jianqiang Xu

**Affiliations:** 1 Liaoning Key Laboratory of Chemical Additive Synthesis and Separation (CASS), School of Chemical Engineering, Ocean Technology and Life Science (CEOTLS) & Panjin Institute of Industrial Technology (PIIT), Dalian University of Technology, Panjin 124221, China; 2 Yingkou Institute of Technology, College of Chemistry and Environmental Engineering, Yingkou 115014, China; 3 State Key Laboratory of Fine Chemicals, School of Chemical Engineering, Dalian University of Technology, Dalian 116023, China

**Keywords:** thioredoxin reductase 1 (TXNRD1), PK11007, autophagy, thioredoxin (TXN1), selenoprotein, p53

## Abstract

Selenoprotein thioredoxin reductase 1 (TXNRD1) is frequently upregulated in various cancer cells to sustain cellular redox homeostasis, and its inhibition has emerged as a promising anti-cancer strategy. In this study, we identified PK11007, a thiol-modifying compound previously characterized as a p53 reactivator, as a potent inhibitor of TXNRD1. PK11007 irreversibly inhibited recombinant TXNRD1 in a time- and dose-dependent manner. Using differential scanning fluorimetry (DSF) and LC–MS/MS analysis, we confirmed that PK11007 covalently modifies the C-terminal redox motif (Cys^497^-Sec^498^) of TXNRD1. In non-small cell lung cancer (NSCLC) H1299 cells, PK11007-induced TXNRD1 inhibition disrupted cellular redox balance, leading to impaired autophagy flux and cell death. Similar autophagy suppression was observed in TXNRD1-knockdown cells, as well as pharmacological inhibition of TXNRD1 by Auranofin (AF) and TXNRD1 inhibitor 1 (TRi-1). Taken together, these findings highlight that oxidative stress contributes to the cytotoxic effects of PK11007 and uncover autophagy disorder as a downstream consequence of TXNRD1 inhibition.

## 1. Introduction

Selenoprotein thioredoxin reductase 1 (TXNRD1) is a key antioxidant enzyme in cells [1,2,3]. Its physiological function is to maintain the reduced state of several thioredoxin-domain containing proteins, such as thioredoxin (TXN1) and thioredoxin-related protein of 14 kDa (TRP14) [4,5,6]. Through this role, TXNRD1 contributes to a broad spectrum of cellular processes, including DNA synthesis, repair, and the regulation of redox-sensitive signaling pathways [7,8,9,10]. TXNRD1 is frequently upregulated in a variety of human cancers and supports tumor cell survival under oxidative stress [11,12,13]. Consequently, TXNRD1 has emerged as a promising therapeutic target in redox-directed strategies [14,15,16].

In recent years, several TXNRD1 inhibitors have been identified [17,18,19,20]. Auranofin, an anti-rheumatic drug, irreversibly inhibits TXNRD1 by modifying its redox-active center [21,22]. TXNRD1 inhibitor 1 (TRi-1) selectively inhibits TXNRD1 through its selenocysteine residue, showing potent cytotoxic efficacy [23,24,25]. Moreover, other small molecules targeting TXNRD1, such as Glaucocalyxin A (Glau A), promote cancer cell death through mechanisms like disulfide stress induced by glutathione depletion [26,27]. Collectively, these studies underline that TXNRD1 inhibition can lad to diverse biological outcomes depending on the cellular context.

PK11007, a low-weight molecule initially developed as an electrophilic p53 reactivator, possesses a covalently reactive group and structural similarities to TRi-1 [28,29]. Intriguingly, several electrophilic p53 reactivators, such as APR-246 (Eprenetapopt) and arsenic trioxide (ATO), exert cytotoxic effects even in p53-deficient cells, specifically through covalent modification of the redox-active TXNRD1, thereby interfering with intracellular redox homeostasis [30,31,32,33,34]. Previous studies have shown that PK11007 induces reactive oxygen species (ROS) production and inhibits cell viability in H1299 cells [29]. However, its precise mechanisms of cytotoxicity in NSCLC cells remain incompletely understood. Therefore, we sought to investigate the cellular effects of PK11007 and the underlying mechanism, focusing on its inhibition of TXNRD1 and the downstream cellular consequences.

In this study, we employed three non-small cell lung cancer (NSCLC) cell lines to compare the cellular responses to PK11007. In H1299 cells, PK11007 treatment induced redox imbalance, triggered phosphorylation of eIF2α, suppressed autophagic flux, and led to cell death. Our findings reveal TXNRD1 inhibition as a key mediator of PK11007-induced cytotoxicity and highlight autophagy dysregulation as a downstream consequencex of TXNRD1 inhibition.

## 2. Materials and Methods

### 2.1. Key Resources

Chemicals: PK11007 (Cat# S89197), TRi-1 (Cat# S87818), Auranofin (Cat# S80655), Ferrostatin-1 (Fer-1, Cat# S81461), Z-VAD-FMK (Cat# S81415) and 5,5′-dithiobis-(2-nitrobenzoic acid) (DTNB, Cat# S19139) were purchased from Yuanye BioTech (Shanghai, China); Necrostatin-1 (Nec-1, Cat# HY-15760, MCE, Shanghai, China); 5-hydroxy-1,4-naphthoquinone (juglone, Cat# H47003, Sigma-Aldrich, St. Louis, MO, USA); 9,10-phenanthrene quinone (9,10-PQ, Cat# P106382, Aladdin BioTech, Shanghai, China); N-acetyl-L-cysteine (NAC, Cat# A601127, Sangon Biotech, Shanghai, China).

Commercial reagents: 0.1% crystal violet (Cat# G1063), and polybrene (Cat# H8761) were acquired from Solarbio (Beijing, China); RPMI-1640 medium (Cat# PM150110) and fetal bovine serum (FBS, Cat# 164210) were obtained from Procell (Wuhan, China); penicillin-streptomycin (P/S, Cat# AC03L332) was obtained from Life-iLab (Shanghai, China); 0.25% Trypsin (Cat# 452321, Sperikon, Chengdu, China); RIPA buffer (Cat# BL504A, Biosharp, Beijing, China); TRIzol reagent (Cat# B511311, Sangon Biotech, Shanghai, China), SYBR Green qPCR Master Mix (SM143, Seven Biotek, Beijing, China).

Plasmids: pLV3-U6-TXNRD1(human)-shRNA1-CopGFP-Puro (Cat# P44479) was purchased from Miaoling BioTech (Wuhan, China).

Proteins: Bovine insulin (Cat# S12033) was purchased from Yuanye BioTech (Shanghai, China). BSA (Cat# NA8692) was obtained from Ruibio (Beijing, China); Recombinant rat TXNRD1 (16.8 U/mg) and recombinant human TXN1 were in-house produced as previously described [35].

Cancer cell lines: H1299 cells (CL-0165), H23 cells (CL-0397), and A549 cells (CL-0016) were obtained from Procell BioTech (Wuhan, China).

Antibodies: anti-TXNRD1 (Cat# 67728), GAPDH (Cat# 60004), FSP1 (Cat# 68049), BAX (Cat# 60267), TXN (Cat# 66475), Vinculin (Cat# 66305), p53 (Cat# 60283), p-eIF2α (Cat# 68023), *p62/SQSTM1* (Cat# 66184), LC3B (Cat# 14600), γ-H2AX (Cat# 83307-2), GPX4 (Cat# 67763), goat anti-mouse IgG (Cat# SA00001-1) and goat anti-rabbit IgG (Cat# SA00001-2) were purchased from Proteintech (Wuhan, China); anti-HSP90 (Cat# R24635), and p-CHK1 (Cat# R381223) were obtained from Zen-Bio (Chengdu, China); anti-HO-1 (Cat# 43996S) was obtained from Cell Signaling Technology (Danvers, MA, USA); anti-CHK1 (Cat# CPA1227) was obtained from Cohesion Biosciences (Bedford, UK).

Critical assays: BCA protein kit (Cat# P0012) was obtained from Beyotime (Shanghai, China); Bradford protein kit (Cat# 20202ES76) was acquired from Yeasen (Shanghai, China); MightyScript^TM^ Plus Reverse Transcriptase kit (Cat# B639252) was purchased from Sangon (Shanghai, China); the ECL mixture solution (Cat# ED0016) was obtained from Sparkjade (Jinan, China); the NAP-5^TM^ column was obtained from Cytiva (Marlborough, MA, USA).

### 2.2. Cell Culture Conditions

H1299 cells, H23 cells, and A549 cells were cultured in RPMI-1640 medium supplemented with 10% FBS, 100 U/mL penicillin, and 100 mg/mL streptomycin (complete RPMI-1640 medium) in a humidified incubator (Heal Force, Shanghai, China) with an atmosphere of 5% CO_2_ at 37 °C.

### 2.3. Cell Viability Assay

Cells were seeded into 96-well plates at a density of 3000 cells per well within 100 μL complete RPMI-1640 medium. Following overnight culture, the cells were treated with various concentrations (0–50 μM) of PK11007 and PK11000 for 24 h.

After treatments, cells were incubated for an additional 2–4 h in 0.5 mg/mL MTT solution in fresh medium, and the intracellular formazan crystals were solubilized with 100 μL DMSO. Absorbance at 570 nm (primary) and 630 nm (background reference) was measured by using the SpectraMax ABS microplate reader (Molecular Devices, San Jose, CA, USA). Cell viability was normalized to the untreated controls and expressed as percentages.

### 2.4. Cell Proliferation Assay

Cells were first treated with 0.25% (*m*/*v*) trypsin to generate single-cell suspensions. Then the cells were seeded in 6-well plates at a density of 1500 cells per well within 2 mL complete RPMI-1640 medium, followed by overnight incubation. After cells were treated with 0, 1, 3 μM PK11007 for 24 h, the medium was replaced, and cells were cultured for 7 days to allow colony formation. Colonies were fixed with 4% (*v*/*v*) paraformaldehyde for 30 min at room temperature, washed twice with PBS, and then stained with 0.1% (*m*/*v*) crystal violet for 15 min. The colony area was calculated by the open-source image processing program ImageJ (1.54g).

### 2.5. Western Blotting

Cells were seeded in 6-well plates and allowed to adhere overnight. After treatment with PK11007 and cell death inhibitors for 12 h, cells were washed with 1.5 mL of cold PBS two times and lysed with RIPA buffer containing 1 mM protease inhibitor PMSF. Cell lysates were centrifuged at 18,000× *g* (1.5 mL × 12, H1850R, Cence, Changsha, China) at 4 °C for 30 min and the soluble supernatants were transferred to new tubes. Protein concentrations of the small aliquots were quantified by using the BCA assay.

Protein samples were mixed with 5 × SDS loading buffer (with 100 mM DTT) and then denatured at 95 °C for 10 min. Afterwards, protein samples were loaded at equal protein amounts onto SDS-PAGE gels. After electrophoresis, the proteins on the gels were transferred to 0.45 μm PVDF membranes. Membranes were blocked with 5% (*m*/*v*) skim milk at room temperature for 1 h, and incubated separately with corresponding primary antibodies (anti-TXNRD1 1:10,000, anti-GAPDH 1:10,000, anti-FSP1 1:5000, anti-GPX4 1:2500, anti-Bax 1:10,000, anti-HSP90 1:2000, anti-HO-1 1:2000, anti-TXN 1:5000, anti-Vinculin 1:5000, anti-p53 1:5000, anti-p-eIF2α 1:5000, anti-p62 1:5000, anti-LC3 1:1000) at 4 °C overnight. After washing with TBS-T (0.1% Tween-20) for three times, membranes were incubated with secondary antibodies (1:10,000) at room temperature for 1 h. Membranes were developed using the ECL mixture solution and chemiluminescent signals were documented by MiniChemi^®^ 610 Chemiluminescent Systemt (Sagecreation, Beijing, China).

### 2.6. Cellular TXNRD Activity Determination

Cellular TXNRD activity was determined according to the end-point insulin-coupled TXN1 reduction assay [36]. Briefly, cell lysates were incubated at 37 °C for 30 min with a reaction mixture containing 80 mM HEPES (pH 7.5), 15 μM TXN1, 300 μM insulin, 660 μM NADPH, and 3 mM EDTA. Reactions without TXN1 served as background controls. The enzymatic reaction was terminated by adding 6 M guanidine hydrochloride supplemented with 1 mM DTNB and 10 mM EDTA, and the absorbance at 412 nm was measured immediately. TXNRD activity was normalized to total protein concentration determined by BCA assay and expressed as percentages.

### 2.7. Recombinant TXNRD1 Activity Determination

The enzymatic activity of recombinant TXNRD1 and its mutants was assessed as previously described [35].

(1) DTNB reduction (10 nM wild-type TXNRD1 or 30–100 nM mutant TXNRD1, 2.5 mM DTNB, 300 μM NADPH in TE buffer, pH 7.5), monitoring the TNB^−^ formation at 412 nm (ε_TNB_ = 13,600 M^−1^ cm^−1^) for 3 min.
(2) 9,10-PQ/juglone reduction (30 nM TXNRD1, 30 μM 9,10-PQ/juglone, 200 μM NADPH), tracking NADPH oxidation at 340 nm (ε_NAPDH_ = 6200 M^−1^ cm^−1^) for 30 min.

All reactions were conducted at 25 °C in a Tecan Infinite 200 PRO microplate reader, with enzyme-free reactions serving as controls.

### 2.8. Glutathione Reductase Activity Assay

Glutathione reductase (GSR) activity was determined by the GSSG reduction assay [37]. GSR was pre-reduced by 100 μM NADPH (10 min, room temperature), and then incubated with PK11007 for 1 h. The activity of GSR was measured by monitoring NADPH oxidation at 340 nm (ε_NAPDH_ = 6200 M^−1^ cm^−1^) in a 200 μL reaction system containing 1 mM oxidized glutathione (GSSG), 200 μM NADPH, and 2 nM yeast GSR in TE buffer (pH 7.5). Activity values were calculated based on NADPH consumption rates, with enzyme-free controls used for background subtraction.

### 2.9. Mass Spectrometry Analysis

To evaluate the modification of PK11007 on TXNRD1, 2 μM pre-reduced TXNRD1 was incubated with 100 μM PK11007 at room temperature (21 ± 1 °C) for 4 h in TE buffer (pH 7.5). Samples were then desalted using NAP-5^TM^ columns (Cytiva, Sweden) and directly subjected to reduction and alkylation. Proteins were denatured and reduced with 20 mM DTT at 65 °C for 1 h, followed by alkylation of free thiols with 40 mM iodoacetic acid (in dark, RT, 30 min). The reaction was quenched by adding 10 mM DTT, and the proteins were digested with trypsin (37 °C, 18 h) in 50 mM ammonium bicarbonate.

Peptides were separated on a Hypersil GOLD™ C18 column (100 × 2.1 mm, 3 μm, Thermo Fisher, Waltham, MA, USA) using a 2–95% (*v*/*v*) acetonitrile/0.1% (*v*/*v*) formic acid gradient (0.4 mL/min, 10 min). Full MS scans (200–2500 *m*/*z*, resolution 70,000, AGC 3e6) and data-dependent MS/MS scans (HCD, collision energy 30, isolation width 4.0 *m*/*z*) were performed on a Thermo Scientific Orbitrap mass spectrometer. MS/MS spectra were acquired at a resolution of 17,500 (max injection time 50 ms, AGC 1e5) [38].

### 2.10. Real-Time PCR

Total RNA was extracted from cells using TRIzol^TM^ reagent and reverse-transcribed into cDNA with the MightyScript^TM^ Plus Reverse Transcriptase Kit (Sangon Biotech, Shanghai, China) following the manufacturer’s protocol. qPCR amplification was conducted on a CG-05 real-time quantitative PCR thermal cycler (Heal-Force, Shanghai, China) using SYBR Green qPCR Master Mix (Thermo Fisher, Waltham, MA, USA).

The primers were synthesized (Sangon Biotech, Shanghai, China) and used in this study as follows: *p62/SQSTM1* forward primer: 5′-TGTGTAGCGTCTGCGAGGGAAA-3′, (22 nt); *p62/SQSTM1* reverse primer: 5′-AGTGTCCGTGTTTCACCTTCCG-3′, (22 nt); *beclin-1* forward primer: 5′-CTGGACACTCAGCTCAACGTCA-3′, (22 nt); and *beclin-1* reverse primer: 5′-CTCTAGTGCCAGCTCCTTTAGC-3′, (22 nt).

### 2.11. Stable Cell Line Generation

HEK 293T cells were transfected with the vector, along with the packaging plasmids psPAX2 and pMD2.G, using PEI-mediated transfection. After 48-h, the virus-containing supernatant was collected, filtered through a 0.45 μm membrane, and supplemented with polybrene at a final concentration of 8 μg/mL to enhance transduction efficiency. The target cells were then incubated with the virus-containing medium. At 48-h post-transduction, the medium was replaced with fresh culture medium containing puromycin (2 μg/mL) for selection. After 7-day selection, stable cell lines were verified by Western blotting using the corresponding antibodies.

### 2.12. Differential Scanning Fluorimetry (DSF) Assay

The DSF assay was performed as previously described [39,40]. 3 μM TXNRD1 was incubated with 100 μM NADPH and 100 μM compounds, including PK11007, APR-246, MQ and TRi-1 separately in a DSF buffer (20 mM HEPES, 100 mM NaCl, pH 7.4) at room temperature for 1 h. Subsequently, SYPRO Orange stain (S6650, Thermo Fisher, Waltham, MA, USA) was added to achieve a final concentration of 5×. The fluorescence signal was recorded using the CG-05 fluorescence spectrometer (Heal-Force, Shanghai, China) while the temperature was increased from 35 °C to 85 °C at a ramp rate of 1 °C/min. The collected fluorescence data were then exported and analyzed using DSFworld (https://gestwickilab.shinyapps.io/dsfworld/ (accessed on 21 July 2025) to determine the apparent melting temperature (Tma) values.

### 2.13. Human Cancer Cell Line Datasets

Human cancer cell line datasets were obtained from the DepMap (The Cancer Dependency Map Project at Broad Institute) (https://depmap.org/portal, accessed on 21 July 2025).

### 2.14. Statistical Analysis

All experiments were independently repeated at least three times, with data presented as mean ± SD (n = 3). Statistical differences between two groups were analyzed using a two-tailed unpaired Student’s *t*-test, while multiple group comparisons were assessed by one-way ANOVA followed by Scheffe’s post hoc test. Significance levels were defined as * *p* < 0.05, ** *p* < 0.01, *** *p* < 0.001, and “n.s.” indicating no significance.

## 3. Results

### 3.1. Electrophilic p53 Re-Activators MQ and PK11007 Inhibit Selenoprotein TXNRD1

**Figure 1:**
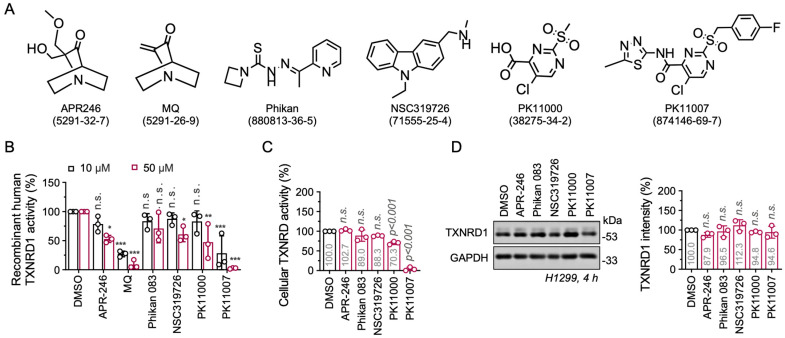
Electrophilic p53 re-activators MQ and PK11007 are inhibitors of TXNRD1. (**A**) Chemical structures of six p53 reactivators. (**B**) Inhibition of recombinant human TXNRD1 by six compounds separately. Black bars represent 10 µM treatments of compounds. (**C**) Cellular TXNRD activity in H1299 cells treated with the indicated compounds. Activities were measured according to end-point insulin-coupled TXN1 reduction assay. (**D**) Western blot analysis of TXNRD1 protein levels in H1299 cells treated with the indicated compounds at 50 µM for 4 h (Left). The TXNRD1 band intensity was normalized to the GAPDH bands and quantified as a percentage in contrast to the DMSO control (Right). Data are presented as mean ± SD (n = 3). Significant differences between groups were evaluated by one-way ANOVA (**C**,**D**), * *p* < 0.05, ** *p* < 0.01, *** *p* < 0.001, and n.s., not significant.

To determine whether TXNRD1 inhibition is a general property of p53 reactivators, we first investigated the interaction between TXNRDs and six selective p53 reactivators, including APR-246 (eprenetapopt), methylene quinuclidinone (MQ), Phikan 083, NSC319726, PK11000, and PK11007 (Figure 1A) [41]. We included MQ as a positive control, as it is a known TXNRD1 inhibitor [31]. Following 1–h incubation at the indicated concentrations, MQ and PK11007 significantly inhibited recombinant TXNRD1 activity, reducing its activity by approximately 90% at 50 µM, whereas Phikan 083 and NSC319726 displayed minimal inhibitory effects (Figure 1B and Figure S1A). Notably, none of these compounds inhibited TXNRD2 (Figure S1B).

Under cellular conditions, PK11007 markedly suppressed cellular TXNRD1 enzymatic activity after 4-h treatment, without affecting its protein expression level (Figure 1C,D), suggesting that PK11007 targets TXNRD1 by inhibiting enzyme activity rather than altering protein expression. Taken together, these results identify PK11007 as a TXNRD1 inhibitor by direct binding with the enzyme.

### 3.2. PK11007 Irreversibly Inhibits TXNRD1 Activity

**Figure 2:**
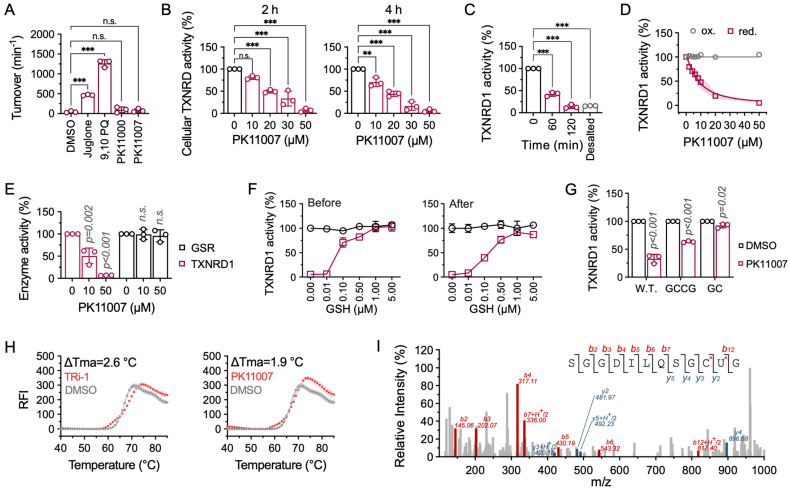
PK11007 inhibits TXNRD1 activity through direct conjugation with the selenocysteine and cysteine residues of TXNRD1. (**A**) NADPH consumption assay showed that neither PK11000 nor PK11007 serves as a redox-cycling inhibitor of TXNRD1. (**B**) Dose-dependent inhibition of cellular TXNRD1 activity by PK11007 treatment. H1299 cells were treated with PK11007 for 2 h and 4 h, respectively. Cells were harvested and the cellular TXNRD1 activity was measured. (**C**) Irreversible inhibition of TXNRD1 by PK11007. NADPH-reduced TXNRD1 (0.2 μM) was incubated with PK11007 (10 μM) for the indicated time and the residual TXNRD1 activity was measured by DTNB reducing activity assay. (**D**) PK11007 inhibited the NADPH-reduced TXNRD1 and the residual activities were analyzed by using the DTNB reduction assay. (**E**) Inhibitory effect of PK11007 on GSR. (**F**) The GSH competition assay was used to assess PK11007’s binding reversibility. Before: Co-incubation of GSH with TXNRD1; After: GSH addition post PK11007-TXNRD1 incubation. Data were normalized to GSH-untreated controls. (**G**) Inhibitory effect of 10 µM PK11007 on WT TXNRD1 and its mutant variants. (**H**) DSF analysis of TXNRD1 treated with 100 μM TRi-1 or PK11007. ΔTm values were calculated relative to vehicle control. (**I**) LC–MS/MS analysis of tryptic peptides of TXNRD1 treated with PK11007 (the red asterisk indicates the modified residues). Data represent as mean ± SD (n = 3). Statistical significance was determined using one-way ANOVA with multiple comparisons (** *p* < 0.01, *** *p* < 0.001, and n.s., not significant).

To further elucidate the mechanism of PK11007-mediated TXNRD1 inhibition, we tested whether PK11007 functions as a redox-cycling inhibitor, like juglone or menadione [42,43]. However, unlike classical TXNRD1 substrates, neither PK11000 nor PK11007 could accept electrons from TXNRD1 (Figure 2A). Instead, PK11007 inhibits both endogenous and recombinant TXNRD1 in a dose-dependent manner (Figure 2B and Figure S2A,B), whereas PK11000 did not exhibit comparable inhibitory effects. Moreover, the inhibition of TXNRD1 by PK11007 is time-dependent and irreversible, as it cannot be rescued by desalting (Figure 2C and Figure S2D).

We then examined whether the oxidation state could influence the inhibition. Results showed that PK11007 inhibited NADPH-reduced TXNRD1 more effectively than the oxidized form (Figure 2D). We next assessed the binding specificity of PK11007 by comparing its effect on glutathione reductase (GSR), which shares sequence homology with TXNRD1. Our results further showed that PK11007 exhibited significantly lower inhibitory activity against GSR than TXNRD1, suggesting its specificity for the C-terminal selenocysteine-containing domain of TXNRD1 (Gly^496^–Cys^497^–Sec^498^–Gly^499^, termed the “GCUG” motif) (Figure 2E and Figure S2C).

In addition, pre-incubation of recombinant TXNRD1 with 0.1 μM glutathione (GSH) significantly preserved enzymatic activity upon PK11007 treatment, resulting in nearly twice the activity compared to post-treatment conditions (Figure 2F). This finding suggests that GSH pre-treatment can transiently shield the redox-active cysteine/selenocysteine residues of TXNRD1 from covalent modification by the thiol-reactive group of PK11007, further supporting its preferential targeting of the reduced state of the enzyme. TXNRD1 mutants, including the Sec-to-Cys mutant, showed reduced sensitivity to PK11007 (Figure 2G and Figure S2E,G), supporting its binding to the C-terminal motif.

Finally, differential scanning fluorimetry (DSF) analysis revealed that PK11007 increased the apparent melting temperature (Tma) of TXNRD1, similar to TRi-1 but distinct from APR-246 and MQ (Figure 2H and Figure S2F).

### 3.3. PK11007 Covalently Binds C-Terminal Redox-Active Residues of TXNRD1

Next, we confirmed the modification of TXNRD1 by PK11007 using LC–MS/MS analysis. We identified a precursor ion of a tryptic peptide from PK11007-treated TXNRD1, exhibiting a calculated *m*/*z* of 827.01 (charge state +2), corresponding to a molecular mass of 1652.02 Da. This peptide is 450.59 Da higher than that of the wild-type TXNRD1 Sec^498^-containing peptide, –SGGDILQSGCUG–, which has a theoretical mass of 1201.43 Da. The observed mass shift of 449.59 Da closely matches the addition of two functional groups of PK11007 (each with a molecular mass of 224.70 Da), suggesting dual covalent modification (Figure 2I and Figure S2H).

MS/MS analysis of the modified peptide further confirmed that both Cys^497^ and Sec^498^ residues were covalently modified by PK11007 (Figure 2I and Figure S2H). This result revealed that PK11007 irreversibly inhibits TXNRD1 activity through covalent modification at the C-terminal redox motif.

### 3.4. Inhibition of TXNRD1 by PK11007 Induces Cell Death in NSCLC Cells

**Figure 3:**
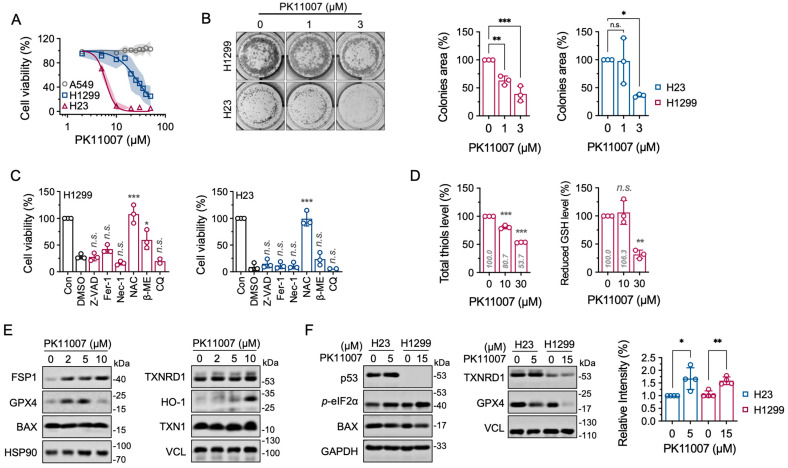
PK11007 induces a ROS-dependent cell death and independent of p53. (**A**) Dose-dependent cytotoxicity of PK11007 in NSCLC cell lines. A549, H1299, and H23 cells were treated with increasing concentrations of PK11007, and cell viability was measured after 24-h treatment. (**B**) Colony formation assay in H1299 and H23 cells treated with PK11007 at 0, 1 and 3 μM for 24 h. Representative images of colony formation are shown. (**C**) Cell death in H1299 (left) and H23 (right) cells treated with PK11007 (10 μM) with or without the indicated concentrations of Z-VAD-FMK, Fer-1, Nec-1, NAC, β-ME and CQ for 24 h. Cell viability was measured and normalized to the DMSO control. (**D**) Total thiols and reduced GSH level in H1299 cells treated with PK11007 for 4 h. (**E**) Western blot analysis of ferroptosis-related and apoptosis-related proteins in H1299 cells treated with PK11007 at the indicated concentrations for 12 h. (**F**) Western blot analysis of indicated proteins in H23 and H1299 cells treated with PK11007 for 12 h. Quantification of p-eIF2α levels normalized to GAPDH is shown (on the very right). Data represent as mean ± SD (n = 3). Statistical significance was determined using one-way ANOVA with multiple comparisons (* *p* < 0.05, ** *p* < 0.01, and *** *p* < 0.001, and n.s., not significant).

To investigate the effect of PK11007 on tumor cells, we assessed its cytotoxic effect by using three NSCLC cell lines such as A549 (p53 wild-type), H23 (p53-mutant), and H1299 (p53-null) cell lines (Figure 3A). Clearly, A549 cells are resistant to PK11007-induced cell death, while the other two cell lines show significant sensitivity. Clonogenic assays further confirmed that PK11007 markedly suppresses colony formation in both H23 (p53-mutant) and H1299 (p53-null) cells in a dose-dependent manner (Figure 3B), implying that the effects of PK11007 are p53-independent.

To elucidate the mechanism underlying PK11007-induced cell death, we employed a panel of regulated cell death inhibitors, including Z-VAD-FMK (apoptosis inhibitor), Fer-1 (ferroptosis inhibitor), Nec-1 (necroptosis inhibitor), NAC (ROS scavenger), β-ME (reducing agent), and chloroquine (CQ, autophagy inhibitor) (Figure 3C). Among these, NAC treatment conferred the strongest protection, indicating that ROS accumulation and integrated stress is the primary driver of PK11007-induced cell death.

### 3.5. Inhibition of TXNRD1 by PK11007 Disrupts Thiol Redox Homeostasis

To further characterize the redox variations caused by PK11007, we measured intracellular total thiol levels. PK11007 treatment significantly decreased both total thiol content and reduced glutathione (GSH) levels in H1299 cells (Figure 3D), indicating the disruption of thiol redox homeostasis. These findings, together with the unaltered TXNRD1 and TXN1 protein levels (Figure 3E, right), suggest that the observed redox imbalance arises from enzymatic inhibition of TXNRD1 rather than changes in protein expression.

Additionally, PK11007 induced a dose-dependent increase in HO-1 protein expression (Figure 3E), consistent with activation of the oxidative stress-like response. Notably, the protein levels of the pro-apoptotic marker BAX remained unchanged (Figure 3E, left), and pan-caspase inhibitor Z-VAD-FMK failed to rescue cell viability (Figure 3C), indicating that PK11007 triggers cell death and might not be related to apoptosis.

To examine whether p53 status influences these stress responses, we compared H23 (mutant p53) and H1299 (p53-null) cells. TXNRD1 protein levels remained constant in both lines, but H1299 cells showed mildly down regulation of GPX4 (Figure 3F, right). Furthermore, upon PK11007 exposure, we observed an increase in eIF2α phosphorylation in both H1299 and H23 cells (Figure 3F, left), suggesting activation of the integrated stress response.

### 3.6. Inhibition of TXNRD1 by PK11007 Inhibits Autophagic Flux

**Figure 4:**
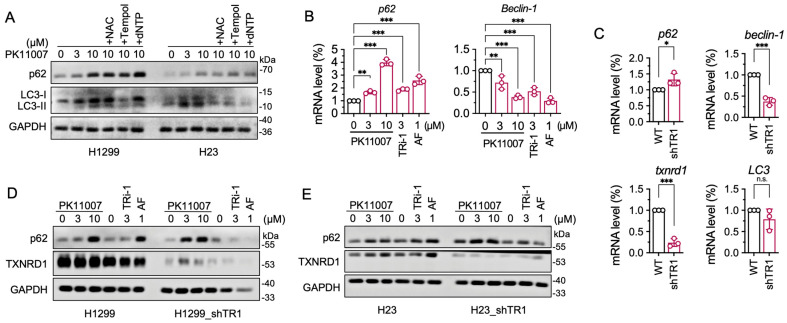
PK11007 suppresses the autophagic flux in H1299 and H23 cells. (**A**) Western blot analysis of autophagy-related protein p62 and LC3 in H1299 and H23 cells treated with 10 μM PK11007 coupled with indicated compounds (NAC, Tempol, and dNTP) for 12 h. (**B**) mRNA levels of p62 and beclin1 in H1299 cells treated with PK11007 or TXNRD1 inhibitors (TRi-1, AF) for 16 h. (**C**) mRNA expression levels of p62, beclin1, LC3, and txnrd1 in H1299 cells and H1299_shTR1 cells, respectively. (**D**) Western blot analysis of p62 and TXNRD1 in H1299 cells and H1299_shTR1 cells treated with PK11007 or TXNRD1 inhibitors (TRi-1, AF) for 12 h. (**E**) Western blot analysis of p62 and TXNRD1 in H23 cells and H23_shTR1 cells treated with PK11007 or TXNRD1 inhibitors (TRi-1, AF) for 12 h. Data represent as mean ± SD (n = 3). Statistical significance was determined using one-way ANOVA with multiple comparisons (* *p* < 0.05, ** *p* < 0.01, *** *p* < 0.001, and n.s., not significant).

We then assessed whether PK11007 impairs autophagy as a downstream event. PK11007 treatment led to p62 accumulation and increased LC3-II/LC3-I ratio in both H1299 and H23 cells (Figure 4A), indicating a blockade of autophagic flux. This was further supported by increased mRNA levels of *SQSTM1* (p62) (Figure 4B). Of note, the PK11007-induced accumulation of LC3-II was alleviated by the ROS scavenger NAC (Figure 4A), implying that the autophagy inhibition is linked to cellular stress induced by PK11007 (Figure S4A,B).

To evaluate the role of TXNRD1 during PK11007 treatment, we generated TXNRD1-knockdown cells (H1299_shTR1 and H23_shTR1). In these cells, mRNA expression of p62 was increased, while beclin1 was downregulated (Figure 4C). In H1299_shTR1 cells, PK11007 treatment significantly increased p62 protein level at 3 μM, indicating that the autophagy pathway was already partially inhibited due to TXNRD1 knockdown (Figure 4D,E). The data above support a model in which PK11007 inhibits TXNRD1 activity, leading to ROS-driven oxidative stress, impaired autophagy, and cancer cell death.

### 3.7. TXNRD1 Inhibition Reduces LC3-II Accumulation Induced by Chloroquine (CQ)

**Figure 5:**
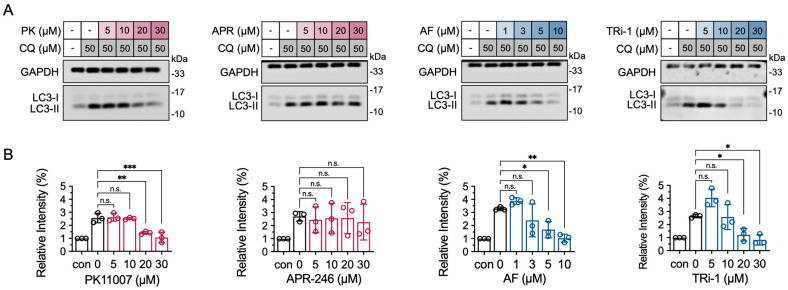
TXNRD1 inhibitors AF and TRi-1 reduce autophagic flux in the presence of chloroquine (CQ). (**A**) Western blot analyses of LC3 in H1299 cells at 5 h post treatment, chloroquine alone or in combination with the indicated doses of PK11007, TRi-1, AF and APR-246, respectively. (**B**) Quantification of LC3-II levels normalized to GAPDH. Data represent as mean ± SD (n = 3). Statistical significance was determined using one-way ANOVA with multiple comparisons (* *p* < 0.05, ** *p* < 0.01, *** *p* < 0.001, and n.s., not significant).

To further investigate the regulatory mechanism of PK11007 on autophagy, we conducted functional validation using the autophagy inhibitor chloroquine (CQ). Interestingly, CQ reversed the LC3-II accumulation induced by TXNRD1 inhibition (Figure 5A,B), suggesting that PK11007-mediated suppression of autophagy is not due to impaired autophagosome–lysosome fusion but rather to interference with autophagosome formation at the initiation stage [44,45]. Interestingly, though both APR-246 and PK11007 are electrophilic p53 reactivators, APR-246 fails to exhibit any notable inhibition on autophagy, which might attribute to a rather weak inhibition of APR-246 on TXNRD1.

## 4. Discussion

In this study, we explored the cellular function of PK11007 in NSCLC cell lines and showed TXNRD1 as its cellular target. Given that TXNRD1 plays a central role in maintaining intracellular redox homeostasis [46,47,48], we further demonstrated that PK11007 induces stress-related cell death by covalently modifying the C-terminal redox-active residues of TXNRD1.

While prior reports have associated TXNRD1 with various forms of programmed cell death, including apoptosis and ferroptosis [49,50,51,52,53,54,55], the mechanism of cell death by TXNRD1 inhibitors remains unclear. Notably, PK11007-induced cell death in H1299 cells is not mediated by classical cell death pathways [56,57]. One proposed mechanism of cancer cell death induced by TXNRD1 inhibition is SecTRAPs (selenium compromised thioredoxin reductase-derived apoptotic proteins) [58,59]. In this study, PK11007-modified TXNRD1 displayed rather low NADPH oxidase activity and did not induce severe apoptotic cell death. Therefore, it remains uncertain whether PK11007 triggers SecTRAPs formation. Instead, our study supports a multifactorial model where TXNRD1 inhibition and glutathione (GSH) depletion act synergistically to disturb cellular redox homeostasis. PK11007, as an electrophilic compound, likely depletes intracellular GSH either through covalent conjugation or by promoting GSSG export, thereby exacerbating the redox imbalance initiated by TXNRD1 inhibition. This synergistic mechanism is consistent with findings from previous studies, such as the work by Wang et al., where TXNRD1 inhibition combined with GSH depletion was shown to be critical for promoting disulfide stress and subsequent cytotoxicity in gastric cancer cells [27].

We further explored the downstream consequences of TXNRD1 inhibition and discovered a link to autophagy regulation. Inhibition of TXNRD1 by PK11007 blocks LC3-II turnover and induces p62 accumulation, indicating impaired autophagic flux. This phenotype was recapitulated using multiple TXNRD1 inhibitors and was validated by TXNRD1 knockdown using shRNA. Furthermore, we observed that inhibition of TXNRD1 by PK11007 suppressed LC3-II accumulation induced by chloroquine, suggesting that TXNRD1 inhibition impairs early autophagy initiation rather than autophagosome-lysosome fusion. These results support a model where TXNRD1 regulates autophagy possibly through redox-sensitive regulators like VPS34 or ATG4 [9,44,60]. However, the contribution of autophagy inhibition in PK11007-induced cell death is uncertain. Our data strongly suggest that oxidative stress is critical for PK11007-induced cell death. The observation of autophagy inhibition is possibly triggered by the increase in the ROS level.

NSCLC frequently exhibits high TXNRD1 expression and many NSCLC cell lines harbor p53 mutations, which makes them particularly relevant to the focus of this study. Interestingly, among the tested NSCLC cell lines, A549 cells displayed relative resistance to PK11007. This resistance can be attributed to the constitutive activation of the NRF2 antioxidant pathway, which is caused by a loss-of-function mutation in KEAP1, the main negative regulator of NRF2 [2]. As a result, hyperactive NRF2 signaling provides strong antioxidant defense, counteracting PK11007-induced oxidative stress and thereby reducing its cytotoxic efficacy.

**Figure 6:**
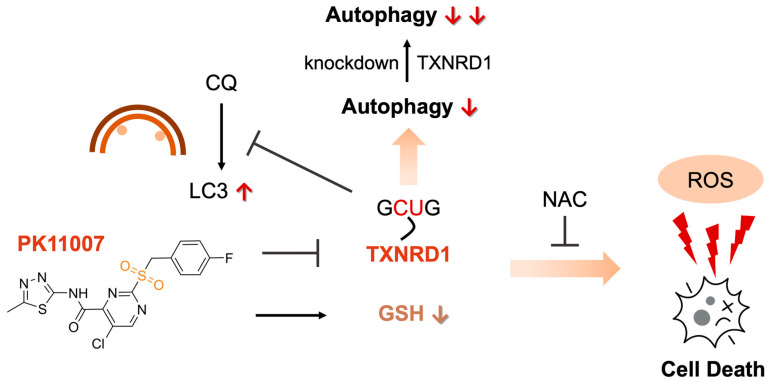
A proposed mechanism of PK11007 in p53-deficient NSCLC cell lines, showing that TXNRD1 inhibition induced by PK11007 treatment may result in autophagy suppression and redox imbalance.

In summary, our findings demonstrate that PK11007 exerts its cellular effects in NSCLC cells through direct inhibition of TXNRD1. This inhibition leads to disruption of TXN-dependent redox homeostasis, oxidative stress, and impaired autophagic flux (as summarized in Figure 6). This model explains its potent cytotoxic activity in NSCLC cells and provides a mechanistic basis for further development of TXNRD1-targeted therapies. Future research should aim to dissect the relative contributions of disulfide stress, autophagy dysregulation, and alternative redox-sensitive pathways in mediating the cytotoxic effects of TXNRD1 inhibitors like PK11007.

## Data Availability

The raw data supporting the conclusions of this article will be made available by the authors on request.

## Supplementary Materials

The following supporting information can be downloaded at: https://www.mdpi.com/2076-3921/14/10/1222/s1, Figure S1: Electrophilic p53 reactivators are inhibitors of TXNRD1 but not of TXNRD2; Figure S2: PK11007 inhibits TXNRD1 activity through direct conjugation at Sec^498^ residue of TXNRD; Figure S3: PK11007 exhibits stronger cytotoxicity than other p53 reactivators in both p53-null and mutant NSCLC cells; Figure S4: PK11007 treatment did not lead to replication stress in tumor cells; Orignial blots.

## References

[1] Reich H.J., Hondal R.J. (2016). Why Nature Chose Selenium. ACS Chem. Biol..

[2] Lennicke C., Cochemé H.M. (2021). Redox Metabolism: ROS as Specific Molecular Regulators of Cell Signaling and Function. Mol. Cell.

[3] Arnér E.S.J. (2009). Focus on Mammalian Thioredoxin Reductases--Important Selenoproteins with Versatile Functions. Biochim. Biophys. Acta.

[4] Andor A., Mohanraj M., Pató Z.A., Úri K., Biri-Kovács B., Cheng Q., Arnér E.S.J. (2023). TXNL1 Has Dual Functions as a Redox Active Thioredoxin-like Protein as Well as an ATP- and Redox-Independent Chaperone. Redox Biol..

[5] Martí-Andrés P., Finamor I., Torres-Cuevas I., Pérez S., Rius-Pérez S., Colino-Lage H., Guerrero-Gómez D., Morato E., Marina A., Michalska P. (2024). TRP14 Is the Rate-Limiting Enzyme for Intracellular Cystine Reduction and Regulates Proteome Cysteinylation. EMBO J..

[6] Meng Y., Sun S., Wang G., Liu H., Shi W., Zhang Y., Wang Z., Zhao J., Liu H., Yang Z. (2025). Mutational Analysis of TXNRD1 Reveals the Essential Role of Trp114 in TRP14 Reduction and Identifies Key Determinants of Enzymatic Activity and Thermostability. Free Radic. Biol. Med..

[7] Kritsiligkou P., Rand J.D., Weids A.J., Wang X., Kershaw C.J., Grant C.M. (2018). Endoplasmic Reticulum (ER) Stress-Induced Reactive Oxygen Species (ROS) Are Detrimental for the Fitness of a Thioredoxin Reductase Mutant. J. Biol. Chem..

[8] Hao X., Zhao B., Towers M., Liao L., Monteiro E.L., Xu X., Freeman C., Peng H., Tang H.-Y., Havas A. (2024). TXNRD1 Drives the Innate Immune Response in Senescent Cells with Implications for Age-Associated Inflammation. Nat. Aging.

[9] Oka S.-I., Chin A., Park J.Y., Ikeda S., Mizushima W., Ralda G., Zhai P., Tong M., Byun J., Tang F. (2020). Thioredoxin-1 Maintains Mitochondrial Function via Mechanistic Target of Rapamycin Signalling in the Heart. Cardiovasc. Res..

[10] Dagnell M., Schmidt E.E., Arnér E.S.J. (2018). The A to Z of Modulated Cell Patterning by Mammalian Thioredoxin Reductases. Free Radic. Biol. Med..

[11] Johnson F.D., Ferrarone J., Liu A., Brandstädter C., Munuganti R., Farnsworth D.A., Lu D., Luu J., Sihota T., Jansen S. (2022). Characterization of a Small Molecule Inhibitor of Disulfide Reductases That Induces Oxidative Stress and Lethality in Lung Cancer Cells. Cell Rep..

[12] McLoughlin M.R., Orlicky D.J., Prigge J.R., Krishna P., Talago E.A., Cavigli I.R., Eriksson S., Miller C.G., Kundert J.A., Sayin V.I. (2019). TrxR1, Gsr, and Oxidative Stress Determine Hepatocellular Carcinoma Malignancy. Proc. Natl. Acad. Sci. USA.

[13] Shi W., Sun S., Liu H., Meng Y., Ren K., Wang G., Liu M., Wu J., Zhang Y., Huang H. (2024). Guiding Bar Motif of Thioredoxin Reductase 1 Modulates Enzymatic Activity and Inhibitor Binding by Communicating with the Co-Factor FAD and Regulating the Flexible C-Terminal Redox Motif. Redox Biol..

[14] Bjørklund G., Zou L., Wang J., Chasapis C.T., Peana M. (2021). Thioredoxin Reductase as a Pharmacological Target. Pharmacol. Res..

[15] Yoo M.-H., Xu X.-M., Carlson B.A., Gladyshev V.N., Hatfield D.L. (2006). Thioredoxin Reductase 1 Deficiency Reverses Tumor Phenotype and Tumorigenicity of Lung Carcinoma Cells. J. Biol. Chem..

[16] Mandal P.K., Schneider M., Kölle P., Kuhlencordt P., Förster H., Beck H., Bornkamm G.W., Conrad M. (2010). Loss of Thioredoxin Reductase 1 Renders Tumors Highly Susceptible to Pharmacologic Glutathione Deprivation. Cancer Res..

[17] Ge Y., Ge Z., Tian F., Tai X., Chen D., Sun S., Shi Z., Yin J., Wei G., Li D. (2024). Sulforaphane Potentiates the Efficacy of Chemoradiotherapy in Glioblastoma by Selectively Targeting Thioredoxin Reductase 1. Cancer Lett..

[18] Chu Y., Nie Q., Zhou X., Yang J., Fang J., Zhang J. (2025). Berberrubine as a Novel TrxR Inhibitor Enhances Cisplatin Sensitivity in the Treatment of Non-Small Cell Lung Cancer. Bioorg. Chem..

[19] Zhang J., Li X., Han X., Liu R., Fang J. (2017). Targeting the Thioredoxin System for Cancer Therapy. Trends Pharmacol. Sci..

[20] Gencheva R., Arnér E.S.J. (2022). Thioredoxin Reductase Inhibition for Cancer Therapy. Annu. Rev. Pharmacol. Toxicol..

[21] Skos L., Schmidt C., Thomas S.R., Park M., Geiger V., Wenisch D., Bonsignore R., Del Favero G., Mohr T., Bileck A. (2024). Gold-Templated Covalent Targeting of the CysSec-Dyad of Thioredoxin Reductase 1 in Cancer Cells. Cell Rep. Phys. Sci..

[22] Yan X., Zhang X., Wang L., Zhang R., Pu X., Wu S., Li L., Tong P., Wang J., Meng Q.H. (2019). Inhibition of Thioredoxin/Thioredoxin Reductase Induces Synthetic Lethality in Lung Cancers with Compromised Glutathione Homeostasis. Cancer Res..

[23] Cheff D.M., Huang C., Scholzen K.C., Gencheva R., Ronzetti M.H., Cheng Q., Hall M.D., Arnér E.S.J. (2023). The Ferroptosis Inducing Compounds RSL3 and ML162 Are Not Direct Inhibitors of GPX4 but of TXNRD1. Redox Biol..

[24] Sabatier P., Beusch C.M., Gencheva R., Cheng Q., Zubarev R., Arnér E.S.J. (2021). Comprehensive Chemical Proteomics Analyses Reveal That the New TRi-1 and TRi-2 Compounds Are More Specific Thioredoxin Reductase 1 Inhibitors than Auranofin. Redox Biol..

[25] Stafford W.C., Peng X., Olofsson M.H., Zhang X., Luci D.K., Lu L., Cheng Q., Trésaugues L., Dexheimer T.S., Coussens N.P. (2018). Irreversible Inhibition of Cytosolic Thioredoxin Reductase 1 as a Mechanistic Basis for Anticancer Therapy. Sci. Transl. Med..

[26] Sun S., Zhang Y., Xu W., Yang R., Yang Y., Guo J., Ma Q., Ma K., Zhang J., Xu J. (2022). Plumbagin Reduction by Thioredoxin Reductase 1 Possesses Synergy Effects with GLUT1 Inhibitor on KEAP1-Mutant NSCLC Cells. Biomed. Pharmacother..

[27] Wang L., Sun S., Liu H., Zhang Q., Meng Y., Sun F., Zhang J., Liu H., Xu W., Ye Z. (2024). Thioredoxin Reductase Inhibition and Glutathione Depletion Mediated by Glaucocalyxin A Promote Intracellular Disulfide Stress in Gastric Cancer Cells. FEBS J..

[28] Synnott N.C., Bauer M.R., Madden S., Murray A., Klinger R., O’Donovan N., O’Connor D., Gallagher W.M., Crown J., Fersht A.R. (2018). Mutant P53 as a Therapeutic Target for the Treatment of Triple-Negative Breast Cancer: Preclinical Investigation with the Anti-P53 Drug, PK11007. Cancer Lett..

[29] Bauer M.R., Joerger A.C., Fersht A.R. (2016). 2-Sulfonylpyrimidines: Mild Alkylating Agents with Anticancer Activity toward P53-Compromised Cells. Proc. Natl. Acad. Sci. USA.

[30] Ceder S., Eriksson S.E., Cheteh E.H., Dawar S., Corrales Benitez M., Bykov V.J.N., Fujihara K.M., Grandin M., Li X., Ramm S. (2021). A Thiol-Bound Drug Reservoir Enhances APR-246-Induced Mutant P53 Tumor Cell Death. EMBO Mol. Med..

[31] Peng X., Zhang M.-Q.-Z., Conserva F., Hosny G., Selivanova G., Bykov V.J.N., Arnér E.S.J., Wiman K.G. (2013). APR-246/PRIMA-1MET Inhibits Thioredoxin Reductase 1 and Converts the Enzyme to a Dedicated NADPH Oxidase. Cell Death Dis..

[32] Liu D.S., Duong C.P., Haupt S., Montgomery K.G., House C.M., Azar W.J., Pearson H.B., Fisher O.M., Read M., Guerra G.R. (2017). Inhibiting the System xC-/Glutathione Axis Selectively Targets Cancers with Mutant-P53 Accumulation. Nat. Commun..

[33] Hedström E., Eriksson S., Zawacka-Pankau J., Arnér E.S.J., Selivanova G. (2009). P53-Dependent Inhibition of TrxR1 Contributes to the Tumor-Specific Induction of Apoptosis by RITA. Cell Cycle.

[34] Lu J., Chew E.-H., Holmgren A. (2007). Targeting Thioredoxin Reductase Is a Basis for Cancer Therapy by Arsenic Trioxide. Proc. Natl. Acad. Sci. USA.

[35] Xu J., Croitoru V., Rutishauser D., Cheng Q., Arnér E.S.J. (2013). Wobble Decoding by the Escherichia Coli Selenocysteine Insertion Machinery. Nucleic Acids Res..

[36] Arnér E.S., Holmgren A. (2001). Measurement of Thioredoxin and Thioredoxin Reductase. Curr. Protoc. Toxicol..

[37] Xu J., Arnér E.S.J. (2012). Pyrroloquinoline Quinone Modulates the Kinetic Parameters of the Mammalian Selenoprotein Thioredoxin Reductase 1 and Is an Inhibitor of Glutathione Reductase. Biochem. Pharmacol..

[38] Zhang H., Cao D., Cui W., Ji M., Qian X., Zhong L. (2010). Molecular Bases of Thioredoxin and Thioredoxin Reductase-Mediated Prooxidant Actions of (-)-Epigallocatechin-3-Gallate. Free Radic. Biol. Med..

[39] Sun S., Liu H., Shi W., Zhou H., Wu H., Xu W., Xu J. (2024). Protocol for Assaying Irreversible Inhibitors of Thioredoxin Reductase 1. STAR Protoc..

[40] Wu T., Hornsby M., Zhu L., Yu J.C., Shokat K.M., Gestwicki J.E. (2023). Protocol for Performing and Optimizing Differential Scanning Fluorimetry Experiments. STAR Protoc..

[41] Peuget S., Zhou X., Selivanova G. (2024). Translating P53-Based Therapies for Cancer into the Clinic. Nat. Rev. Cancer.

[42] Sun S., Xu W., Zhang Y., Yang Y., Ma Q., Xu J. (2021). Menadione Inhibits Thioredoxin Reductase 1 via Arylation at the Sec498 Residue and Enhances Both NADPH Oxidation and Superoxide Production in Sec498 to Cys498 Substitution. Free Radic. Biol. Med..

[43] Xu J., Cheng Q., Arnér E.S.J. (2016). Details in the Catalytic Mechanism of Mammalian Thioredoxin Reductase 1 Revealed Using Point Mutations and Juglone-Coupled Enzyme Activities. Free Radic. Biol. Med..

[44] Pérez-Pérez M.E., Zaffagnini M., Marchand C.H., Crespo J.L., Lemaire S.D. (2014). The Yeast Autophagy Protease Atg4 Is Regulated by Thioredoxin. Autophagy.

[45] Swamynathan M.M., Kuang S., Watrud K.E., Doherty M.R., Gineste C., Mathew G., Gong G.Q., Cox H., Cheng E., Reiss D. (2024). Dietary Pro-Oxidant Therapy by a Vitamin K Precursor Targets PI 3-Kinase VPS34 Function. Science.

[46] Prasad C.B., Oo A., Liu Y., Qiu Z., Zhong Y., Li N., Singh D., Xin X., Cho Y.-J., Li Z. (2024). The Thioredoxin System Determines CHK1 Inhibitor Sensitivity via Redox-Mediated Regulation of Ribonucleotide Reductase Activity. Nat. Commun..

[47] Delgobo M., Gonçalves R.M., Delazeri M.A., Falchetti M., Zandoná A., Nascimento das Neves R., Almeida K., Fagundes A.C., Gelain D.P., Fracasso J.I. (2021). Thioredoxin Reductase-1 Levels Are Associated with NRF2 Pathway Activation and Tumor Recurrence in Non-Small Cell Lung Cancer. Free Radic. Biol. Med..

[48] Wang C., Zhang L., Cao M., Fu Z., Wang H., Zhang S., Zhu K., Hou Z., Cui J., Yue P. (2023). Thioredoxin Facilitates Hepatocellular Carcinoma Stemness and Metastasis by Increasing BACH1 Stability to Activate the AKT/mTOR Pathway. FASEB J..

[49] Liu X., Nie L., Zhang Y., Yan Y., Wang C., Colic M., Olszewski K., Horbath A., Chen X., Lei G. (2023). Actin Cytoskeleton Vulnerability to Disulfide Stress Mediates Disulfidptosis. Nat. Cell Biol..

[50] Tang M., Dirks K., Kim S.Y., Qiu Z., Gao Y., Sun D., Peruggia G., Sallavanti J., Li W. (2025). Inhibition of Thioredoxin Reductase 1 Sensitizes Glucose-Starved Glioblastoma Cells to Disulfidptosis. Cell Death Differ..

[51] Zhong Z., Zhang C., Ni S., Ma M., Zhang X., Sang W., Lv T., Qian Z., Yi C., Yu B. (2023). NFATc1-Mediated Expression of SLC7A11 Drives Sensitivity to TXNRD1 Inhibitors in Osteoclast Precursors. Redox Biol..

[52] Llabani E., Hicklin R.W., Lee H.Y., Motika S.E., Crawford L.A., Weerapana E., Hergenrother P.J. (2019). Diverse Compounds from Pleuromutilin Lead to a Thioredoxin Inhibitor and Inducer of Ferroptosis. Nat. Chem..

[53] Dos Santos A.F., Fazeli G., Xavier da Silva T.N., Friedmann Angeli J.P. (2023). Ferroptosis: Mechanisms and Implications for Cancer Development and Therapy Response. Trends Cell Biol..

[54] Saitoh M., Nishitoh H., Fujii M., Takeda K., Tobiume K., Sawada Y., Kawabata M., Miyazono K., Ichijo H. (1998). Mammalian Thioredoxin Is a Direct Inhibitor of Apoptosis Signal-Regulating Kinase (ASK) 1. EMBO J..

[55] Reynoso E., Liu H., Li L., Yuan A.L., Chen S., Wang Z. (2017). Thioredoxin-1 Actively Maintains the Pseudokinase MLKL in a Reduced State to Suppress Disulfide Bond-Dependent MLKL Polymer Formation and Necroptosis. J. Biol. Chem..

[56] Hadian K., Stockwell B.R. (2023). The Therapeutic Potential of Targeting Regulated Non-Apoptotic Cell Death. Nat. Rev. Drug Discov..

[57] Dixon S.J., Olzmann J.A. (2024). The Cell Biology of Ferroptosis. Nat. Rev. Mol. Cell Biol..

[58] Cheng Q., Antholine W.E., Myers J.M., Kalyanaraman B., Arnér E.S.J., Myers C.R. (2010). The Selenium-Independent Inherent pro-Oxidant NADPH Oxidase Activity of Mammalian Thioredoxin Reductase and Its Selenium-Dependent Direct Peroxidase Activities. J. Biol. Chem..

[59] Anestål K., Prast-Nielsen S., Cenas N., Arnér E.S.J. (2008). Cell Death by SecTRAPs: Thioredoxin Reductase as a Prooxidant Killer of Cells. PLoS ONE.

[60] Nagakannan P., Iqbal M.A., Yeung A., Thliveris J.A., Rastegar M., Ghavami S., Eftekharpour E. (2016). Perturbation of Redox Balance after Thioredoxin Reductase Deficiency Interrupts Autophagy-Lysosomal Degradation Pathway and Enhances Cell Death in Nutritionally Stressed SH-SY5Y Cells. Free Radic. Biol. Med..

