# Supplementary material for: PK11007 Covalently Inhibits Thioredoxin Reductase 1 to Induce Oxidative Stress and Autophagy Impairment in NSCLC Cells
Source: Antioxidants (Basel). 2025 Oct 11;14(10):1222. doi: 10.3390/antiox14101222 (PMC12709224; doi:10.3390/antiox14101222)
Supplement: Supplementary file 1 [file antioxidants-14-01222-s001.zip › antioxidants-3803992-supplementary.pdf]

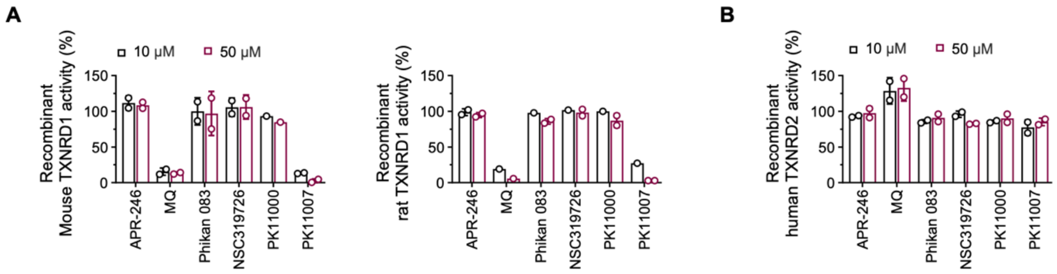

Figure S1. Electrophilic p53 re-activators are inhibitors of TXNRD1 but not of TXNRD2. **(A)** Inhibition of recombinant human TXNRD1 activity by six selected compounds including APR-246, MQ, Phikan 083, NSC319726, PK11000 and PK11007. DTNB reducing activity of TXNRD1 was measured in vitro and normalized to the DMSO control. **(B)** Inhibition of recombinant human TXNRD2 activity by six selected compounds including APR-246, MQ, Phikan 083, NSC319726, PK11000 and PK11007. DTNB reducing activity of TXNRD2 was measured in vitro and normalized to the DMSO control.

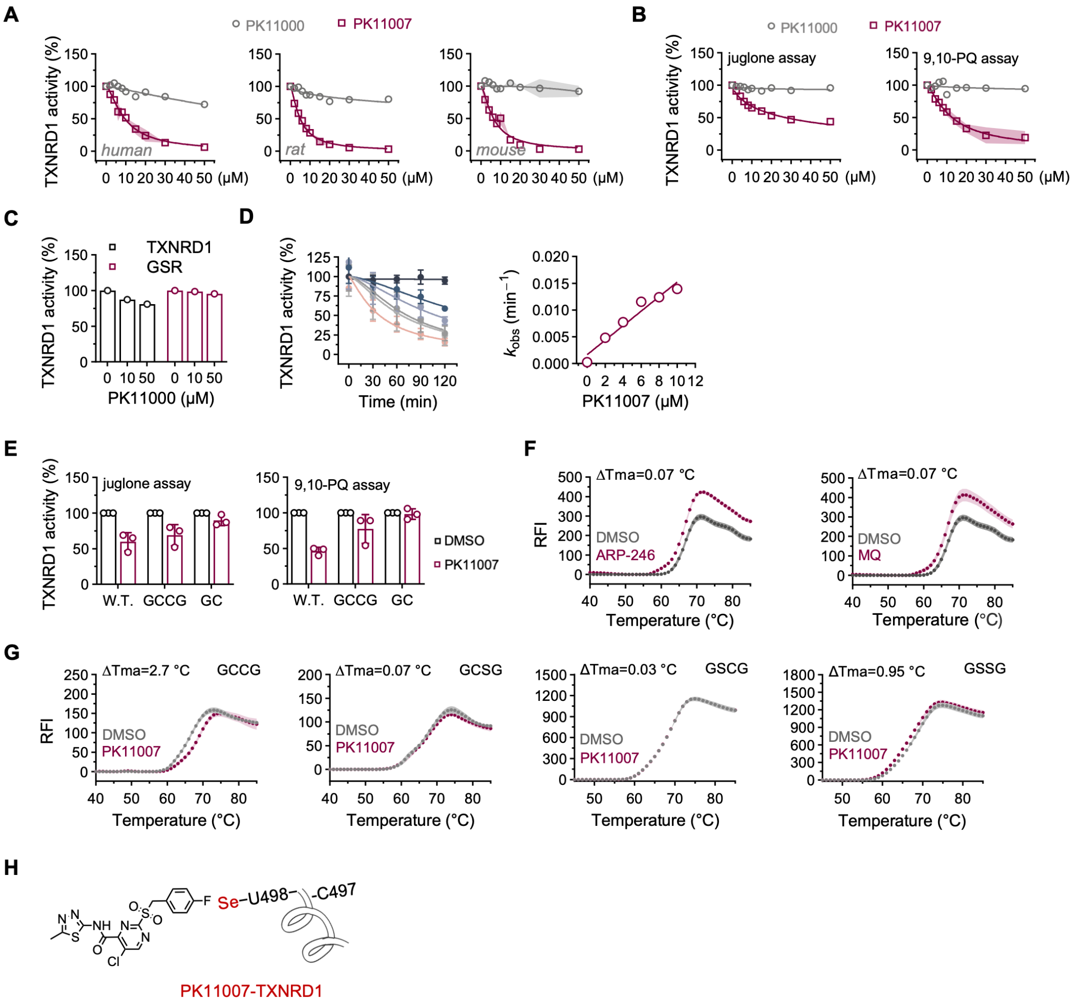

Figure S2. PK11007 inhibits TXNRD1 activity through direct conjugation at Sec498 residue of TXNRD1. **(A)** Dose-dependent inhibition of recombinant human, rat, and mouse TXNRD1 by PK11007 and PK11000. **(B)** Inhibition of TXNRD1 activity by PK11007 and PK11000 using two different substrates, juglone and 9,10-phenanthrenequinone (9,10-PQ). **(C)** PK11000 does not inhibit either TXNRD1 or GSR. **(D)** The time-dependent inhibition of TXNRD1 by PK11007 (the left), as well as the corresponding second-order rate constant (kobs) plotted against PK11007 concentrations (the right), as determined by the DTNB assay, indicate an irreversible inhibition. **(E)** Inhibitory activity of PK11007 against various TXNRD1 mutants, assessed using 9,10-phenanthrenequinone and juglone as substrate. **(F-G)** Differential scanning fluorimetry (DSF) was used to analyze the thermal denaturation of TXNRD1 after incubation with 100 μM APR-246 or MQ (above), and to assess the impact of PK11007 on the thermal stability of various TXNRD1 mutants (bottom). DMSO was used as the control. ΔTm values calculated relative to vehicle control according to the DSFworld software (https://gestwickilab.shinyapps.io/dsfworld/). **(H)** Simulated molecular model of the interaction between PK11007 and TXNRD1.

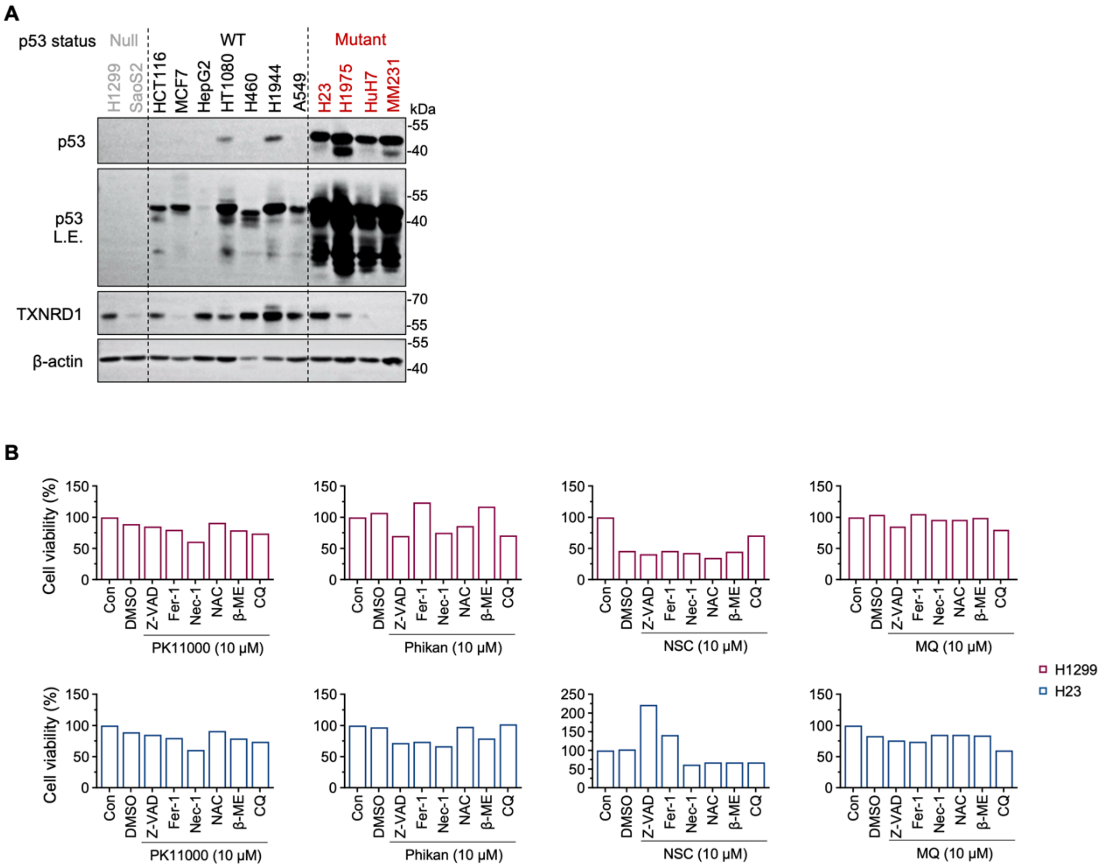

Figure S3. PK11007 exhibits stronger cytotoxicity than other p53 reactivators in both p53-null and mutant NSCLC cells. **(A)** Western blot analysis of p53 and TXNRD1 in human cancer cell lines with different p53 statuses: null (H1299, Saos2), wild-type (HCT116, MCF7, HepG2, HT1080, H460, H1944, A549), and mutant (H23, H1975, Huh7, MM231). L.E.: long exposure. **(B)** Cell death in H1299 and H23 cells treated with indicated five p53 reactivators (10 μM) with or without Z-VAD-FMK, Fer-1, Nec-1, NAC, β-ME and Chloroquine (CQ) at the indicated concentrations for 24 h. Cell viability was measured and normalized to the DMSO control.

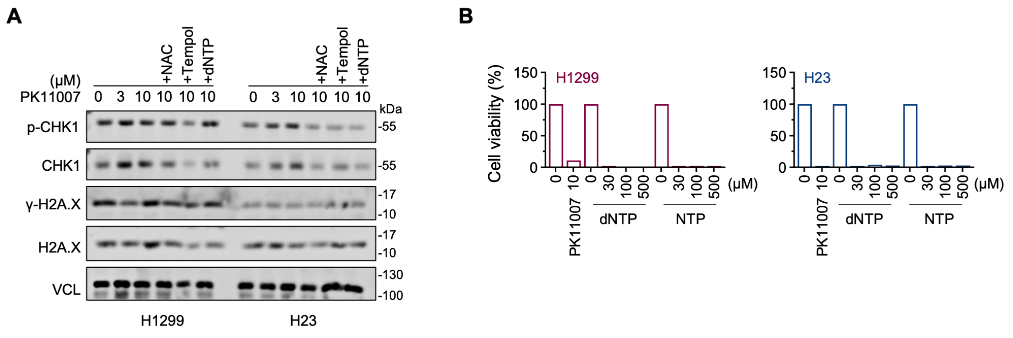

Figure S4. PK11007 treatment did not lead to replication stress in tumor cells. **(A)** Western blot analysis of DNA replication stress markers p-CHK1/CHK1 and γ-H2AX/H2AX in H1299 cells treated with PK11007 coupled with antioxidants (NAC, Tempol, dNTP) for 12 h. **(B)** Cell viability of H1299 and H23 cells treated with PK11007, either alone or in combination with 100 μM deoxyribonucleotides (dNTP) or nucleotides (NTP), at indicated concentrations.
